# Community-based approaches for neonatal survival: meta-analyses of randomized trial data

**DOI:** 10.2471/BLT.16.175844

**Published:** 2017-04-24

**Authors:** Claudia Hanson, Sanni Kujala, Peter Waiswa, Tanya Marchant, Joanna Schellenberg

**Affiliations:** a Department of Public Health Sciences, Karolinska Institutet, Widerströmska huset, Stockholm, 171 77, Sweden.; b London School of Hygiene & Tropical Medicine, London, England.; c School of Public Health, Makerere University, Kampala, Uganda.

## Abstract

**Objective:**

To analyse the impact of community approaches to improving newborn health and survival in low-resource countries.

**Methods:**

We updated previous meta-analyses of published cluster randomized trials of community-based interventions for neonatal survival. For each study we extracted baseline data on the context: geographical area; available facilities and staffing; immediate breastfeeding and facility births; and neonatal mortality. We also extracted data on the primary outcome (neonatal survival) and intermediate outcomes of the interventions (changes in immediate breastfeeding and facility births). We used forest plots and pooled sub-group analysis to seek patterns in associations between the effect size and the context or type of intervention (home-based counselling or women’s groups).

**Findings:**

We included 17 trials, spanning years from 2001 to 2013. A 25% reduction in neonatal mortality (relative risk, RR: 0.75; 95% confidence interval, CI: 0.69–0.80) was found when pooling six studies in settings with 44 or more deaths per 1000 live births. In lower-mortality settings (pooling six studies with 32 or fewer deaths per 1000 live births) there was no evidence of an effect. We observed some evidence that community approaches had a stronger effect in south Asia than in sub-Saharan Africa. Community approaches had a lower impact on neonatal mortality in settings where at least 44% of women delivered in a facility.

**Conclusion:**

As neonatal mortality declined, the impact of community approaches on survival appeared to be lower, and the role of these approaches in supporting newborn care in weak health systems may need to be re-examined.

## Résumé

**Objectif:**

Analyser l'impact des approches communautaires dans l'amélioration de la survie et de la santé des nouveau-nés dans des régions à faibles ressources.

**Méthodes:**

Nous avons actualisé d'anciennes méta-analyses d'essais randomisés en grappes publiés, portant sur des interventions communautaires visant à améliorer la survie néonatale. Pour chaque étude, nous avons extrait les données de référence sur le contexte: zone géographique, centres et effectifs disponibles; proportion de naissances dans un centre de soins et d’initiation immédiate de l'allaitement; mortalité néonatale. Nous avons également extrait les données sur le principal critère étudié (survie néonatale) et les effets indirects des interventions (modification des pratiques en termes d'initiation immédiate de l'allaitement et d'accouchement dans un centre de soins). À l'aide de graphiques en forêt et d'une analyse groupée portant sur les sous-groupes nous avons recherché d'éventuels schémas d'association entre l'importance de l'impact et le contexte ou le type d'intervention (conseils à domicile ou participation à des réunions de groupes de femmes).

**Résultats:**

Nous avons inclus 17 essais, couvrant la période de 2001 à 2013. Une réduction de 25% de la mortalité néonatale (risque relatif: 0,75; intervalle de confiance de 95%: 0,69–0,80) a été observée dans l'analyse groupée de six études réalisées dans des régions dont le taux de mortalité était de 44 décès ou plus pour 1 000 naissances vivantes. Dans les régions au taux de mortalité inférieur (analyse groupée de six études réalisées dans des régions avec un taux de mortalité de 32 décès ou moins pour 1 000 naissances vivantes), aucun effet n'a été constaté. Plusieurs éléments probants montrent que les approches communautaires ont eu un impact plus important en Asie du Sud qu'en Afrique subsaharienne. Les approches communautaires ont eu un impact plus faible sur la mortalité néonatale dans les régions où au moins 44% des femmes accouchaient dans un centre de soins.

**Conclusion:**

À mesure que la mortalité néonatale recule, les approches communautaires sur la survie néonatale semblent avoir moins d'impact. Le rôle de ces approches pour renforcer les soins néonataux dans les régions au système de santé fragile pourrait nécessiter un réexamen.****

## Resumen

**Objetivo:**

Analizar el impacto de enfoques comunitarios para mejorar la salud y la supervivencia de los neonatos en países con escasos recursos.

**Métodos:**

Se actualizaron metaanálisis anteriores de ensayos aleatorizados de conglomerados publicados de intervenciones comunitarias de supervivencia de los neonatos. Para cada estudio, se extrajeron datos de referencia según el contexto: zona geográfica; centros y personal disponibles; lactancia inmediata y nacimientos en centros; y mortalidad neonatal. También se extrajeron datos sobre los resultados primarios (supervivencia neonatal) y los resultados intermedios de las intervenciones (cambios de la lactancia inmediata y los nacimientos en centros). Se utilizaron parcelas y se realizaron análisis de subgrupos en busca de patrones asociativos entre el tamaño del efecto y el contexto o tipo de intervención (asesoría doméstica o grupos de mujeres).

**Resultados:**

Se incluyeron 17 ensayos desde el año 2001 hasta el 2013. Se identificó una reducción del 25% en la mortalidad neonatal (riesgo relativo: 0,75; intervalo de confianza (IC) del 95%: 0,69–0,80) al realizar seis estudios en centros con 44 o más muertes por cada 1 000 nacidos vivos. En lugares con escasa mortalidad (se realizaron seis estudios con 32 muertes o menos por cada 1 000 nacimientos vivos) no se encontraron pruebas de un efecto. Se observaron algunas pruebas de que los enfoques comunitarios lograron un mayor efecto en el sur de Asia que en el África subsahariana. Los enfoques comunitarios tuvieron un menor impacto para la mortalidad neonatal en lugares en los cuales al menos un 44% de las mujeres daban a luz en un centro.

**Conclusión:**

Dado que la mortalidad neonatal cayó, el impacto de los enfoques comunitarios sobre la supervivencia ha sido menor, y puede que sea necesario volver a examinar la función de estos enfoques en el respaldo de la atención para los recién nacidos en sistemas sanitarios pobres.

## ملخص

**الغرض:**

تحليل أثر الأساليب المجتمعية على تحسين صحة حديثي الولادة وبقائهم على قيد الحياة في البلدان منخفضة الموارد.

**الطريقة:**

قمنا بتحديث التحاليل التلوية السابقة للتجارب العشوائية العنقودية المنشورة والمتعلقة بالتدخلات المجتمعية لإبقاء حديثي الولادة على قيد الحياة. وقد استخلصنا البيانات الرئيسية من كل دراسة في هذا المجال: المنطقة الجغرافية؛ والتوظيف والمرافق الصحية المتاحة؛ والرضاعة الطبيعية الفورية ومرافق الولادة؛ ووفيات الأطفال حديثي الولادة. كما استخلصنا بيانات حول النتيجة الأولية (بقاء حديثي الولادة على قيد الحياة) والنتائج المتوسطة للتدخلات (التغييرات في الرضاعة الطبيعية الفورية ومرافق الولادة). و استخدمنا مخططات غابية وتحليل المجموعات الفرعية المجمعة للبحث عن أنماط في العلاقات بين حجم التأثير وسياق التدخل أو نوعه (المشورة المنزلية أو المجموعات النسائية).

**النتائج:**

لقد قمنا بتضمين 17 تجربة، تمتد ما بين عامي 2001 و2013. وتم العثور على انخفاض بنسبة 25‏٪ في وفيات حديثي الولادة (المخاطر النسبية: 0.75؛ وبنسبة أرجحية مقدارها ‏‏95%: 0.69–0.80) عند تجميع ست دراسات في شهدت 44 حالة وفاة أو أكثر لكل 1000 مولود حي. وفي البيئات منخفضة الوفيات (تجميع ست دراسات بها 32 حالة وفاة أو أقل لكل 1000 مولود حي) لم يكن هناك دليل على وجود أي تأثير. ولاحظنا وجود بعض الأدلة على أن الأساليب المجتمعية لها تأثير أقوى في جنوب آسيا منها في مناطق جنوب الصحراء بأفريقيا. كان للأساليب المجتمعية تأثير أقل على وفيات حديثي الولادة في المناطق التي قامت فيها نسبة ‏44‏% من النساء بالولادة في مرافق صحية.

**الاستنتاج:**

ومع انخفاض وفيات حديثي الولادة، يبدو أن للأساليب المجتمعية المتعلقة بالبقاء على قيد الحياة تأثير أقل، كما أنه قد يلزم إعادة النظر في دور هذه الأساليب في دعم رعاية حديثي الولادة في النظم الصحية الضعيفة.

## 摘要

**目的:**

： 旨在分析改善新生儿健康和存活率的社区方法在资源匮乏国家的影响。

**方法：:**

我们更新了之前对公布的基于社区的新生儿存活率干预的群体随机试验数据的元分析。 提取了各项研究在以下方面的基准数据： 地理区域；适用医院和人员；直接母乳喂养和住院分娩；以及新生儿死亡率。 我们还提取了干预（直接母乳喂养和住院分娩方面的改变）的主要成果（新生儿存活率）和间接成果。 我们采用了森林图和汇总亚组分析，以找出效应量与干预背景或类型（基于家庭的指导或产妇分组）之间的关联模式。

**结果:**

： 我们纳入了 2001 年到 2013 年间的 17 项试验。汇总在每 1000 名活产婴儿中有 44 例或更多例死亡的地区开展的六项研究数据之后，我们发现新生儿死亡率降低了 25%（相对危险度： 0.75；95% 置信区间： 0.69–0.80）。 而在死亡率较低的地区（汇总六项研究，每 1000 名活产婴儿中有 32 例或更少例死亡），没有证据表明有成效。 我们观察到的一些证据表明，社区方法在南亚地区比在撒哈拉以南非洲地区的效果更明显。 在至少有 44% 产妇在医院生产的地区，社区方法对新生儿死亡率的影响较小。

**结论：:**

随着新生儿死亡率的降低，社区方法对存活率的影响似乎也减小了。而在卫生体系薄弱的地区，此类方法在支持新生儿护理方面的作用有待重新考察。****

## Резюме

**Цель:**

Провести анализ влияния общинных подходов, направленных на улучшение здоровья и повышение выживаемости новорожденных в странах с низким уровнем ресурсообеспеченности.

**Методы:**

Авторы обновили предыдущие метаанализы опубликованных кластерных рандомизированных исследований вмешательств, подразумевающих широкое участие местного населения, нацеленных на повышение выживаемости новорожденных. Для каждого исследования были извлечены исходные данные о контексте: географическая область, имеющиеся медицинские учреждения и персонал, применение грудного вскармливания непосредственно после родов и роды в медицинском учреждении, неонатальная смертность. Авторы также извлекли данные относительно первичного исхода (выживаемости новорожденных), а также относительно промежуточных результатов вмешательств (изменений в применении грудного вскармливания непосредственно после родов и проведении родовспоможения в медицинском учреждении).Авторы использовали форест-графики и объединенный анализ подгрупп, чтобы найти закономерности во взаимосвязи между размером эффекта и контекстом или типом вмешательства (консультирование на дому или женские группы).

**Результаты:**

Авторы включили 17 испытаний, проведенных в период с 2001 по 2013 год. Было обнаружено снижение неонатальной смертности на 25% (относительный риск: 0,75; 95%-й доверительный интервал, ДИ: 0,69–0,80) при объединении шести исследований в условиях 44 смертей или более на 1000 живорождений. В условиях более низкой смертности (объединение шести исследований с 32 смертями или менее на 1000 живорождений) признаки эффекта обнаружены не были. Авторы наблюдали некоторые признаки того, что общинные подходы оказали более сильный эффект в Южной Азии, чем в Африке к югу от Сахары. Общинные подходы оказали меньшее влияние на неонатальную смертность в тех местах, где как минимум 44% женщин рожали в медицинском учреждении.

**Вывод:**

Поскольку уровень неонатальной смертности снизился, влияние общинных подходов на выживаемость оказалось ниже. Возможно, потребуется пересмотреть роль этих подходов в поддержке медицинского ухода за новорожденными в слабо развитых системах здравоохранения.****

## Introduction

Despite progress in reducing child deaths in the past 25 years, an estimated 2.6 million neonatal deaths occurred globally in 2015.^1^ Sustainable development goal (SDG) 3 included the target of no more than 12 deaths per 1000 live births in the first 28 days of life.^2^ To reach the target, more effective ways of delivering quality preventive and curative care need to be identified and monitored.

Approaches based on health promotion and on community empowerment and participation have long been promoted as part of formal health-care systems in low- and middle-income countries.^3^^,^^4^ Trials to improve maternal and newborn health through community approaches have focused on two approaches: (i) home-based counselling^5^ and (ii) participatory women’s groups.^6^ Both approaches promote appropriate care-seeking as well as improved home practices in newborn care. Home-based counselling focuses on health education and behaviour change to improve newborn care practices by mothers, such as immediate breastfeeding, dry cord care and appropriate health care (e.g. delivering in a health-care facility and seeking care for sick newborns). Women’s groups use an empowerment and problem-solving approach aiming similarly to improve care practices and care-seeking by mothers of newborns. The mechanisms of the effect of the home-based counselling strategies are backed by an analysis using the Lives Saved tool.^7^

Previous meta-analyses have reported moderate effects on neonatal mortality of both home-based counselling and women’s groups. A meta-analysis of five proof-of-principle trials of home-based counselling in south Asia in 2010 found an almost 40% reduced risk of neonatal death (relative risk, RR: 0.62; 95% confidence interval, CI: 0.44–0.87).^5^ In response, the World Health Organization (WHO) recommended home visits to improve neonatal health in high neonatal mortality settings.^8^ However, trials of home-based counselling conducted in a larger population and in programme settings ^9^^,^^10^ showed a smaller risk reduction for neonatal mortality (RR: 0.93; 95% CI: 0.85–1.01).^9^ A review of seven trials of women’s groups based on participatory learning and action cycles published in 2013 reported a 20% reduction in neonatal mortality (RR: 0.77; 95% CI: 0.65–0.90).^6^ The evidence prompted WHO to recommend community mobilization with women’s groups to improve maternal and neonatal health.^11^

Factors reported to have the greatest impact on neonatal mortality include how successfully the intervention was implemented, as reflected by the proportion of pregnant women participating in women’s groups;^6^ the inclusion of injectable antibiotics for treatment of possible severe bacterial infection;^6^ and home management of asphyxia.^5^ However, it is not clear how the women’s group approach works,^12^ or what is the interaction between community approaches and contextual factors, such as the characteristics of the health-care system.

In this paper we updated previous searches and meta-analyses of trials of home-based counselling and women’s groups in low-resource countries. The aim was to generate and test hypotheses about which factors may lead to weaker or stronger effects on neonatal survival. We examined associations between reductions in neonatal mortality and the context in which the trial took place or the characteristics of the local health system. We also assessed associations between reductions in mortality and the characteristics of the implementation.

## Methods

### Inclusion criteria and search methods

**Fig. 1:**
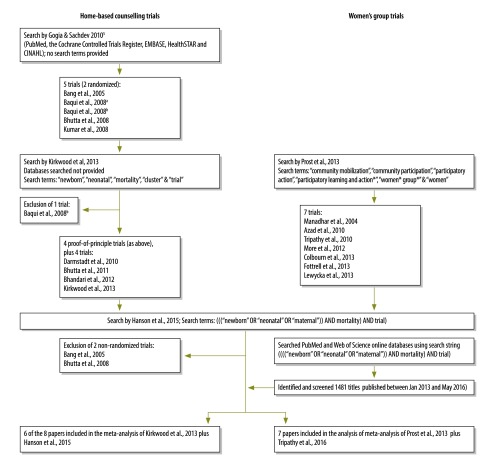
Flowchart showing the selection of articles for meta-analysis of the effect of community approaches for neonatal survival

^a^ Baqui et al.^13^

^b^ Baqui et al.^28^

We reviewed cluster randomized trials evaluating community approaches to enhancing neonatal survival in low- and middle-income countries in April 2016, covering all studies published to this date. All trials compared neonatal mortality in pregnant women receiving the intervention with those receiving the local standard care (Table 1). We included trials of both home-based counselling and facilitated women’s groups delivered during pregnancy. Our starting point was two previously published reviews^5^^,^^10^ of five trials of home-based counselling interventions,^13^^,^^14^^,^^26^^,^^27^ and another five published between 2010 and 2013.^9^^,^^10^^,^^15^^–^^17^ One trial was excluded from the review as it was only quasi-experimental.^28^ We also included a review published in 2013^6^ covering seven trials of women’s groups.^18^^–^^24^ To identify the most recently published trials we conducted a literature search of the PubMed and Web of Science online databases using the following search string ((((“newborn” OR “neonatal” OR “maternal”)) AND mortality) AND trial), and identified trials of home-based counselling or women’s group interventions published between January 2013 and May 2016 in low- or middle-income countries (Fig. 1). We screened 1481 titles and identified one additional cluster randomized trial that examined women’s groups in rural eastern India.^25^ Another identified trial^29^lacked a randomized design and was not included. Although they had been included in earlier meta-analyses by other authors, we excluded two non-randomized trials^26^^,^^27^ from our meta-analysis after an assessment of the risk of bias. 

**Table 1:** Populations, intervention characteristics and intermediate outcomes for randomized cluster studies included in the meta-analysis of community-based approaches for neonatal survival

**Study type and authors**	**Evaluation period**	**Area, country**	**Setting**	**Neonatal deaths in trial area, per 1000 live births**	**Study population, no.**	**Study design^a^**		**Intermediate outcomes^b^**			
						**Intervention**	**No. of clusters in trial**	**Immediate breastfeeding, % of births**		**Facility births, % of births**	
								**Baseline**	**Change**	**Baseline**	**Change**
**Home-based counselling trials**											
Baqui et al., 2008^13^	2003–2005	Sylhet, Bangladesh	Poor rural	44	~ 480 000	Community meetings + home-based counselling visits (2 in pregnancy and 3 postpartum) + home treatment if referral failed	16	45	+28	10	+1
Kumar et al., 2008^14^	2003–2005	Shivgarh, India	Poor rural	84	104 123	Community meetings + home-based counselling visits (2 in pregnancy and 2 postpartum)	26	4	+65	8	+9
Kumar et al., 2008^14^	2003–2005	Shivgarh, India	Poor rural	84	104 123	Community meetings + home-based counselling visits (2 in pregnancy and 2 postpartum) + ThermoSpot^c^	26	3	+63	3	+15
Darmstadt et al., 2010^15^	2005–2006	Mirzapur, Bangladesh	Poor rural	28	292 000	Home-based counselling visits (2 in pregnancy and 4 postpartum)	12	41	+25	12	+4
Bhutta et al., 2011^16^	2006–2008	Hala, Pakistan	Poor rural	49	600 000	Community mobilization + home-based counselling visits (2 in pregnancy and 2 postpartum)	16	27	+16	44	+10
Bhandari et al., 2012^17^	2008–2010	Haryana, India	Poor rural	43	1 100 000	Home-based counselling visits (3 postpartum)	18	11^d^	+30	N/A	N/A
Kirkwood et al., 2013^10^	2008–2009	Newhints, Ghana	Poor rural	32	600 000	Home-based counselling visits (2 in pregnancy and 3 postpartum)	98	41^d^	+7	58	0
Hanson et al., 2015^9^	2010–2013	Mtwara and Lindi, United Republic of Tanzania	Poor rural	30	1 200 000	Home-based counselling visits (3 in pregnancy and 2 postpartum)	132	19	+7	43	+2
**Women’s group trials**											
Manandhar et al., 2004^18^	2001–2003	Makwanpur Nepal	Poor rural	37	400 000	Monthly participatory women’s group meetings	24	54^d^	+8	2^d^	+5
Tripathy et al., 2010^19^	2005–2008	Jharkhand and Orissa, India	Poor rural	60	228 186	Monthly participatory learning + action cycle	36	61^d^	0	20^d^	−6
Azad et al., 2010^20^	2005–2007	Bogra, Bangladesh	Poor rural	38	503 163	Participatory learning + action cycle	18	51	N/A	7	0
More et al., 2012^21^	2006–2009	Mumbai, India	Urban slum	11	282 000	Bi-monthly participatory meetings including peer learning	48	82	0	87^d^	−1
Colbourn et al., 2013 ^d^^,^^22^	2007–2010	MaiKanda, Malawi	Poor rural	34	2 500 000	Monthly participatory learning + action cycle	32	N/A	N/A	41	+17
Colbourn et al., 2013^ e^^,^^22^	2007–2010	Kasungu, Lilongwe and Salima, Malawi	Poor rural	34	2 500 000	Participatory learning + action cycle + facility strengthening	30	N/A	N/A	52	+18
Fottrell et al., 2013^23^	2009–2011	Bogra, Bangladesh	Poor rural	30	532 996	Monthly participatory learning + action cycle	18	65	+7	19	+1
Lewycka et al., 2013^24^	2004–2010	MaiMwana, Malawi	Poor rural	30	185 888	Monthly participatory learning + action cycle, with and without volunteer peer counselling	36	78	+2	36	+9
Tripathy et al., 2016^25^	2009–2012	Jharkhand and Orissa, India	Poor rural	63	156 519	Monthly participatory learning + action cycle	30	77	+1	48	+4

N/A: not available.

^a^ All studies were cluster randomized trials comparing neonatal mortality in the population receiving the intervention with mortality in a comparison population receiving the local standard care.

^b^ Immediate breastfeeding was defined in most studies as the percentage of births in which the infant was breastfed within 1 hour of delivery (mother’s report), except Bhutta et al.^16^ who defined breastfeeding within 30 minutes, and Tripathy et al.^19^ who defined breastfeeding within 4 hours of birth. Facility birth was defined in all studies as the percentage of births in a health-care facility. Baseline was the value at the trial baseline in the intervention and comparison groups. Change was the change in values between the trial baseline and endline separately for intervention and comparison groups (the difference-in-differences).

^c^ ThermoSpot™ (Camborne Consultants, Dorset, England) is a non-invasive liquid crystal indicator for hypothermia.

^d^ For trials that did not report on newborn practices at baseline and endline we did not calculate the difference-in-difference change but the simple difference between estimates from intervention and comparison group.

^e^ The intervention group included all 24 clusters with women’s groups (with and without additional peer counselling). The comparison group included the 12 clusters without any intervention. However, the data on increases in breastfeeding and facility births were calculated with the comparison group of all clusters without women’s groups as no data were available separately for the clusters with no intervention.

Note: This table shows intermediate outcomes but the primary outcome for all studies was population-based neonatal mortality rate obtained either from surveys or continuous surveillance in the target population.

### Data processing

Two authors independently assessed the risk of bias (allocation concealment, and method of data collection for neonatal mortality data) for each study included in the review using the Cochrane Collaboration tool.^30^

For each trial we extracted data on the study context (geographical area; baseline neonatal mortality rate; baseline proportion of births with infant breastfed immediately after delivery; baseline proportion of births in a facility); health system characteristics in the trial area (number of nurses and midwives per 1000 population; number of health facilities per 100 000 population); and type of intervention (home-based counselling or women’s groups). We also extracted data on the strength of the implementation (proportion of pregnant women visited in home-based counselling or attending women’s groups). Not all the variables were reported in all trials. The data were obtained from the published papers and through contacting authors. One author extracted data, which were subsequently checked by another author. We performed all analysis in Stata, version 13.0 (Stata Corp, College Station, United States of America).

The primary outcome for all studies was neonatal mortality. We also used immediate breastfeeding and facility births as tracer indicators for good newborn care practices. We calculated the changes in the proportions of women breastfeeding immediately after delivery and giving birth in a facility between baseline and endline separately for intervention and comparison groups (the difference-in-differences). When baseline figures were not available, we calculated the differences between the intervention and control groups at endline.

### Analysis

We used the *metan* command in STATA to compute forest plots calculating the RR for neonatal mortality for each study based on the number of deaths and births reported in intervention and comparison groups at the end of the trial period. Heterogeneity was assessed and *I^2^* and *P*-values were tabulated together with the summary estimates to provide measures of heterogeneity. We used the forest plots to examine patterns in the effect size on neonatal mortality according to the study context or health system characteristics in the trial area. We also investigated patterns in neonatal survival according to features of the implementation. For the analysis of associations between intervention characteristics and the effect size we chose equal-sized groups. For example, we categorized the 17 trials into three groups based on neonatal mortality rates in the trial area: very high mortality settings (≥ 44 deaths per 1000 live births), high mortality settings (33–43 deaths per 1000 live births) and moderately high mortality settings (≤ 32 deaths per 1000 live births).

## Results

### Included trials

We included 15 articles^9^^,^^10^^,^^13^^–^^25^ reporting 17 trials (two papers^13^^,^^22^ reported two studies each). Eight studies reported interventions using home-based counselling and nine were interventions based on women’s groups.

The trials took place in sub-Saharan Africa (Ghana, 1 trial; Malawi, 3 trials; United Republic of Tanzania, 1 trial) and in south Asia (Bangladesh, 4 trials; India, 6 trials; Nepal, 1 trial; Pakistan, 1 trial). All the trials were done in poor rural societies, except for the trial in an urban slum in India^21^ (Table 1).

All packages aimed to improve home-based newborn care by mothers, such as immediate and exclusive breastfeeding, thermal care, and safe and dry cord care; the home care arm from one study^13^ encouraged home treatment with antibiotics if referral was not possible (Table 1). Most trials reported coverage of these newborn practices as intermediate outcomes. Home-based behaviour change counselling involved visits to pregnant women at home by a community health worker or volunteer and sometimes also included community meetings. Women’s participatory groups took place in the community and were facilitated by trained community members who used problem-solving methods, such as action cycles. Both approaches included education and behaviour change communication to overcome challenges in health-care seeking and home newborn care practices.

All trials reported neonatal mortality as the main outcome, defined as the number of deaths in the first 28 days of life per 1000 live births in both sexes. Neonatal mortality data were obtained either from surveys or continuous surveillance in the target population. The trials were done in diverse contexts where the neonatal mortality rate ranged from 11 deaths per 1000 live births in an Indian urban slum^21^ to 84 deaths per 1000 live births in India.^14^ While the reported trials from Asia were from a period spanning the years 2001 to 2012, the reported trials from sub-Saharan Africa were from the years 2004 to 2013 (Table 1).

### Context characteristics

**Fig. 2:**
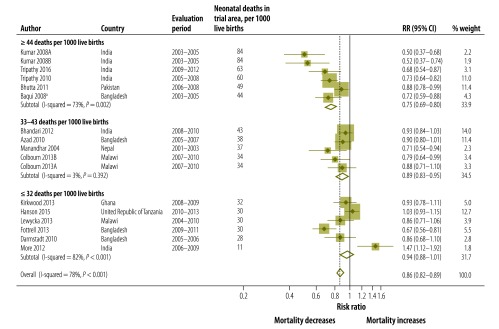
Meta-analysis of the effect on neonatal mortality of trials of community approaches for neonatal survival, by neonatal mortality rate at baseline

CI: confidence interval; RR: relative risk.

Notes: For references with more than one trial, each trial is presented separately and denoted with a letter after the date. Baseline neonatal mortality rate was defined as the number of deaths within 28 days after birth per 1000 live births in the comparison group in the study area.

**Fig. 3:**
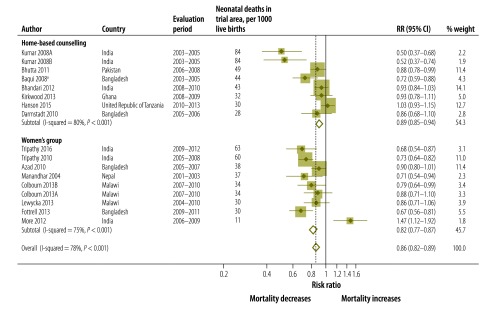
Meta-analysis of the effect on neonatal mortality of trials of community approaches for neonatal survival, by type of approach

CI: confidence interval; RR: relative risk.

^a^ Baqui et al.^13^

Notes: For references with more than one trial, each trial is presented separately and denoted with a letter after the date. Home-based behaviour-change counselling involved home visits to individual pregnant women by a community health worker and sometimes included community meetings. Women’s participatory groups took place in the community and were facilitated by trained community members. Both approaches included education, behaviour change communication and a problem-solving approach to improve newborn care behaviours by mothers, such as immediate and exclusive breastfeeding, thermal care, and safe and dry cord care.

We observed the largest reduction of neonatal mortality in settings with very high neonatal mortality. We calculated a 25% reduction in neonatal mortality (RR: 0.75; 95% CI: 0.69–0.80) when pooling six studies (*P* = 0.002 for heterogeneity) which took place in very high mortality settings of ≥ 44 deaths per 1000 live births. The effect on neonatal mortality was smaller (RR: 0.89; 95% CI: 0.83–0.95) when pooling five trials (*P* = 0.392 for heterogeneity) in areas with high neonatal mortality of 33–43 deaths per 1000 live births, while there was no evidence of an effect on neonatal mortality (RR: 0.94; 95% CI: 0.88–1.01) when pooling six trials (*P* < 0.001 for heterogeneity) in settings with moderately high neonatal mortality of ≤ 32 deaths per 1000 live births (Fig. 2; Table 2). The pattern of the largest reductions in settings with the highest neonatal mortality was observed for both home-based counselling and women’s group approaches (Fig. 3).

**Table 2:** Effect on neonatal mortality of trials of community-based approaches for neonatal survival, stratified by context and implementation characteristics

Stratification variable	No. of trials or trial arms	RR (95% CI) random effects model	Tests for heterogeneity /^2^, %	*P* for heterogeneity in sub-groups
**Neonatal mortality in comparison group, no. of deaths per 1000 live births**				
≤ 32	6	0.94 (0.88–1.01)	82	< 0.001
33–43	5	0.89 (0.83–0.95)	3	0.392
≥ 44	6	0.75 (0.69–0.80)	73	0.002
**Geographical area**				
South Asia	12	0.82 (0.78–0.86)	81	< 0.001
Sub-Saharan Africa	5	0.95 (0.88–1.02)	34	0.193
**Immediate breastfeeding at baseline, % of births**^a,b^				
≤ 25	5	0.91 (0.85–0.98)	87	< 0.001
26–53	4	0.87 (0.81–0.94)	29	0.239
≥ 54	5	0.81 (0.73–0.90)	85	< 0.001
**Facility births at baseline, % of births**^b,c^				
≤ 10	5	0.77 (0.71–0.85)	80	0.001
11–43	6	0.85 (0.80–0.91)	80	< 0.001
≥ 44	5	0.90 (0.83–0.97)	80	0.001
**Density of facilities in study area, no. per 100 000 population**				
≤ 8	5	0.84 (0.78–0.90)	74	< 0.001
> 9	4	0.95 (0.88–1.04)	48	0.121
**Density of nurses and midwives in study area, no. per 1000 population**				
≤ 0.4	4	0.85 (0.79–0.92)	87	< 0.001
> 0.4	2	0.86 (0.73–0.99)	0	0.721
**Type of intervention**				
Home-based counselling	8	0.89 (0.85–0.94)	80	< 0.001
Women’s group	9	0.82 (0.77–0.87)	75	< 0.001
**Immediate breastfeeding, % points change at endline**^d^				
≤ +5	4	0.81 (0.74–0.89)	88	< 0.001
+5 to +24	5	0.90 (0.84–0.96)	79	0.001
≥ +25	5	0.82 (0.76–0.89)	83	< 0.001
**Facility births, % points change at endline**^d^				
≤ +1	6	0.83 (0.78–0.88)	84	< 0.001
+2 to +8	4	0.92 (0.85–1.00)	79	< 0.003
≥ +9	6	0.81 (0.75–0.88)	73	0.002
**Coverage of home-based counselling, % of pregnant women**^e^				
37–66	3	0.92 (0.86–0.99)	81	0.005
≥ 67	5	0.86 (0.79–0.93)	83	< 0.001
**Coverage of women’s groups, % of pregnant women attending**^f^				
≤ 36	5	0.87 (0.81–0.95)	83	< 0.001
37–66	4	0.74 (0.68–0.82)	0	0.418

CI: confidence interval; RR: relative risk.

^a^ Immediate breastfeeding was defined in most studies as the percentage of births in which the infant was breastfed within 1 hour of delivery, except Bhutta et al.^16^ who defined breastfeeding within 30 minutes, and Tripathy et al.^19^ who defined breastfeeding within 4 hours of birth.

^b^ Baseline was the value at the trial baseline (in the intervention and comparison groups).

^c^ Facility birth was defined in all studies as the percentage of births in a health-care facility.

^d^ Change was the change in values between the trial baseline and endline separately for intervention and comparison groups (the difference-in-differences).

^e^ Percentage of pregnant women visited at home by a community health worker.

^f^ Percentage of pregnant women attending their local women’s group.

**Fig. 4:**
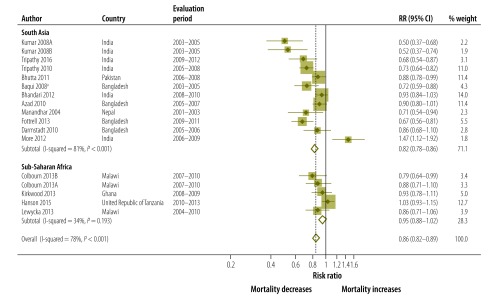
Meta-analysis of the effect on neonatal mortality of trials of community approaches for neonatal survival, by region

CI: confidence interval; RR: relative risk.

^a^ Baqui et al.^13^

Note: For references with more than one trial, each trial is presented separately and denoted with a letter after the date.

The pooled analysis suggested that the effects of the community approaches on neonatal mortality were stronger in the 12 pooled studies in south Asia (RR: 0.82; 95% CI: 0.78–0.86; *P* < 0.001 for heterogeneity), while there was no evidence of an effect in five studies in sub-Saharan Africa (RR: 0.95; 95% CI: 0.88–1.02; *P* = 0.193 for heterogeneity). None of the African studies, however, were done in a setting with very high neonatal mortality (Fig. 4; Table 2).

**Fig. 5:**
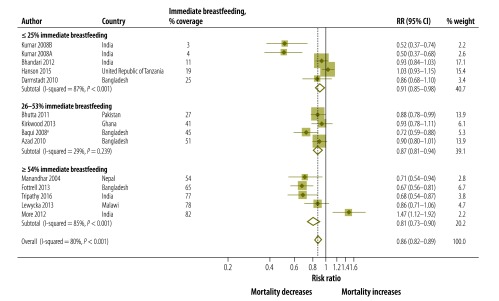
Meta-analysis of the effect on neonatal mortality of trials of community approaches for neonatal survival, by immediate breastfeeding at baseline

CI: confidence interval; RR: relative risk.

^a^ Baqui et al.^13^

Notes: For references with more than one trial, each trial is presented separately and denoted with a letter after the date. Immediate breastfeeding was defined in most studies as the percentage of births in which the infant was breastfed within 1 hour of delivery, except Bhutta et al.^16^ who defined breastfeeding within 30 minutes, and Tripathy et al.^19^ who defined breastfeeding within 4 hours of birth.

**Fig. 6:**
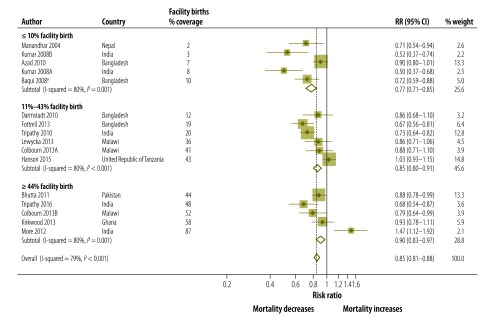
Meta-analysis of the effect on neonatal mortality of trials of community approaches for neonatal survival, by facility births at baseline

CI: confidence interval; RR: relative risk.

^a^ Baqui et al.^13^

Notes: For references with more than one trial, each trial is presented separately and denoted with a letter after the date. Facility birth was defined in all studies as the percentage of births in a health-care facility.

Overall, we did not observe any clear pattern of effects of immediate breastfeeding at baseline on neonatal mortality (Fig. 5; Table 2). However, trials done in settings with very high baseline neonatal mortality had lower rates of immediate breastfeeding (Fig. 5) and of facility births at baseline (Fig. 6). The mean baseline level of immediate breastfeeding was 31% (range 3–77%) in very high mortality settings, 39% (range 11–54%) in high mortality settings and 52% (range 19–82%) in moderately high mortality settings. A similar trend was seen for facility births, whereby levels were 22% (range 3–48%), 26% (range 2–52%) and 43% (range 12–87%) in very high, high and moderately high neonatal mortality settings, respectively. 

The effect size of the community approaches was somewhat higher (RR: 0.77; 95% CI: 0.71–0.85) in pooled data from five studies (*P* = 0.001 for heterogeneity) where the baseline level of facility births was low (≤ 10%). The effect was lower when pooling six studies with 11–43% births in a facility (RR: 0.85; 95% CI: 0.80–0.91; *P* < 0.001 for heterogeneity) and five studies with ≥ 44% facility births (RR: 0.90; 95% CI: 0.83–0.97; *P* < 0.001 for heterogeneity; Fig. 6; Table 2).

Only nine trials reported the health-system characteristics of facilities in the trial area. The meta-analysis suggested a lower effect of the community-based approaches on neonatal mortality in settings with more health facilities (Table 2). No evidence of community approaches was observed (RR: 0.95; 95% CI: 0.88–1.04) when pooling four studies (*P* = 0.121 for heterogeneity) with a density of > 9 facilities per 100 000 population. However, we found a 16% reduction (RR: 0.84; 95% 0.78–0.90) when pooling five studies (*P* < 0.001 for heterogeneity) in areas with ≤ 8 facilities per 100 000 population. Only six trials reported on the number of nurses and midwives in the area and we observed no difference in the effect on neonatal mortality in settings with higher or lower number of nurses and midwives per population (Table 2).

### Implementation characteristics

**Fig. 7:**
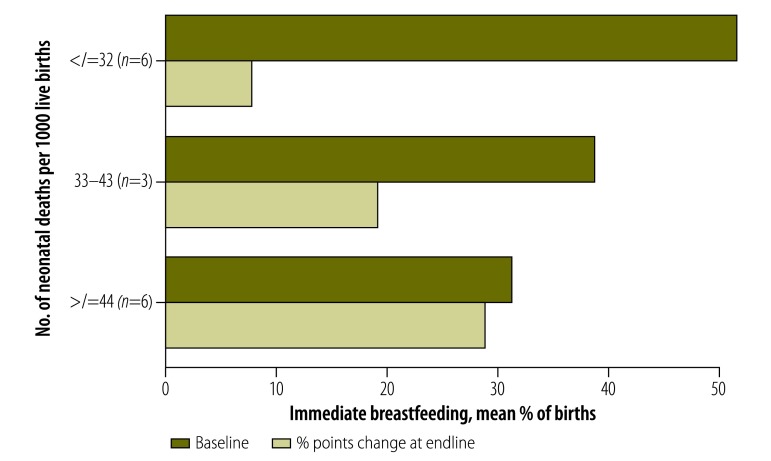
Mean baseline and changes in proportion of women breastfeeding immediately after delivery, by neonatal mortality in trial area

Notes: Immediate breastfeeding was defined in most studies as the percentage of births in which the infant was breastfed within 1 hour of delivery, except Bhutta et al.^16^ who defined breastfeeding within 30 minutes, and Tripathy et al.^19^ who defined breastfeeding within 4 hours of birth.

**Fig. 8:**
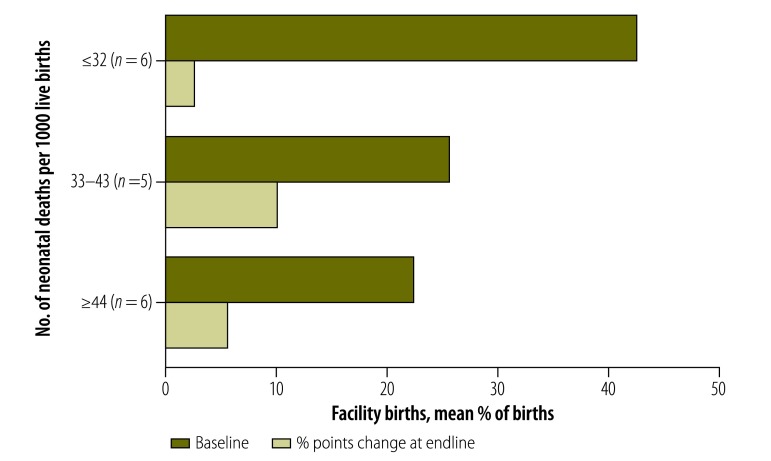
Mean baseline and changes in proportion of women delivering in a facility, by neonatal mortality in trial area

Note: Facility birth was defined in all studies as the percentage of births in a health-care facility.

The mean improvement in immediate breastfeeding was a +29% point change in very high mortality settings, while a change of only +8% points was observed in moderately high mortality settings (Fig. 7; available at: http://www.who.int/bulletin/volumes/95/6/16-175844). The change in facility births was +6% points (range: −6 to 15) in very high mortality settings, +10% points (range: 0 to 18) in high mortality settings and +3% points (range: −1 to 9) in moderately high mortality settings (Fig. 8; available at: http://www.who.int/bulletin/volumes/95/6/16-175844).

*n* is the number of trials.

*n* is the number of trials.

We observed no evidence that the effect of the community-based approaches on neonatal mortality was associated with improvements in immediate breastfeeding and facility births. The analysis pooling five trials which achieved improvement in immediate breastfeeding of 25% or more suggested a reduction of neonatal mortality of 18% (RR: 0.82; 95% CI: 0.76–0.89; *P* < 0.001 for heterogeneity). Similarly, the pooled analysis of four trials achieving only marginal improvement (≤ 5%) in immediate breastfeeding suggested a 19% reduction in neonatal mortality (RR: 0.81; 95% CI: 0.74–0.89; *P* < 0.001 for heterogeneity; Table 2).

**Fig. 9:**
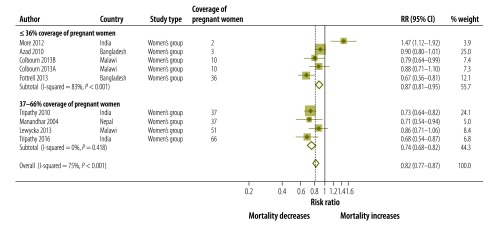
Meta-analysis of the effect on neonatal mortality of community approaches for neonatal survival in women’s group trials, by coverage of pregnant women

CI: confidence interval; RR: relative risk.

Notes: For references with more than one trial, each trial is presented separately and denoted with a letter after the date. Coverage of home-based counselling was the percentage of pregnant women visited at home by community health workers. Coverage of women’s groups was the percentage of pregnant women attending their local women’s group.

All home-based counselling interventions reached more than 40% of pregnant women and the size of the effect of the intervention on neonatal mortality did not differ in relation to the proportion of women reached. However, a difference was seen when running a sub-analysis of the women’s group interventions. Pooling four trials that reached 37–66% of pregnant women we found a 26% reduction in neonatal mortality (RR: 0.74; 95% CI: 0.68–0.82; *P* = 0.418 for heterogeneity). In contrast, pooling the five studies which received < 36% coverage suggested a lower effect size on neonatal mortality (RR: 0.87; 95% CI: 0.81–0.95; *P* < 0.001 for heterogeneity; Fig. 9; Table 2).

## Discussion

Our analysis suggests that large gains in neonatal survival can be achieved using community approaches in settings with very high neonatal mortality and very low rates of facility births. Where mortality is lower, although still moderately high, no evidence of an effect of community approaches on neonatal mortality was found. The observed effect size of the community approaches was larger in south Asia, while there was no evidence of an effect when pooling the studies done in sub-Saharan Africa. This might be partly explained by the fact that the trials in Ghana, Malawi and the United Republic of Tanzania were done in settings with moderately high neonatal mortality.

The large effect of a 45% reduction of neonatal mortality which was previously reported^5^ could be because these early trials were done in settings with high mortality and unhealthy home-care practices. Except in one trial,^25^ subsequent meta-analysis^9^^,^^10^ included trials done in places where neonatal mortality was considerably lower.

As neonatal mortality in an area decreases, the relative importance of infectious diseases and other more easily addressable risk factors, such as cold injuries, reduces. The latest work of the Global Burden of Disease group clearly highlights the increasing importance of intrapartum complications, including neonatal encephalopathy, as causes of death.^31^ As non-infectious causes of neonatal mortality become more prominent, health system constraints to prevent intrapartum-related complications and mitigate the effect of prematurity might become more important.^32^ One study concluded that part of the reason their intervention did not result in mortality reduction ‒ despite improved neonatal care and facility coverage ‒ was the failure to address birth asphyxia and prematurity.^15^ A similar argument was raised by others reporting on community and participatory women’s group approaches.^21^^,^^33^

Lower neonatal mortality is likely to reflect recent or ongoing trends in health service uptake, household wealth education and health literacy. One study reported a decrease in neonatal mortality in both intervention and comparison groups, accompanied by increases in newborn care practices and health-service uptake, suggesting underlying trends that had a larger impact than the trial intervention itself.^20^ Others reported a doubling of facility deliveries during the trial period that was possibly due in part to increased transportation and better communications in the area.^9^ A third study suggested that a reduction in neonatal mortality in both intervention and control groups was likely related to improvements in the living environment in the slum areas, such as covering gutters and better sanitation and electricity supplies.^21^ These welcome investments in the health system and overall development reflect a rapidly changing context in which it is inherently more difficult to show large mortality reductions from specific interventions.^34^

Many of the trials in our analysis reported only a very modest improvement in the numbers of women delivering in a health-care facility. This is in contrast to the most recent large increases in facility births observed in many low- and middle-income countries; these have occurred because of multiple factors, both within and outside their health systems.^35^

Going forward, the strategies and content of community approaches to neonatal survival might need to be re-examined. Still many potential benefits of community approaches to enhancing health literacy, reducing delays in care-seeking and improving linkages between the community and health facilities for emergency referral exist. Community approaches can also encourage accountability measures that could support facility strengthening.^36^However, the effect on neonatal mortality would depend on the quality of services available, and the two effects could not be separated.

Our approach of examining effects of community approaches in relation to context and health-system factors has to be interpreted with caution. We hypothesize that in settings with lower neonatal mortality, more facility births and improved newborn care practices, these interventions may have less effect. However, our stratified meta-analysis cannot prove such an association. Our findings are plausible against the background that the present community approaches target neonatal sepsis and complications of prematurity, while in a context of reduced neonatal mortality, intrapartum-related complications leading to asphyxia become more important.^37^ Reducing intrapartum complications and birth asphyxia will demand quality intrapartum services which the present community approaches do not address.

We combined the two different approaches of home-based counselling and women’s groups in our analysis, which strictly speaking prohibits any meta-analysis. Nevertheless, both approaches aimed to improve mothers’ newborn care practices at home and health-seeking behaviours, and thus the mediators through which they affect neonatal mortality are expected to be similar. Our main aim was not to present summary estimates of the mortality effect to guide policy changes. Rather, we hope to contribute to the development of a theory underpinning the opportunities and limitations of community approaches and the role these approaches might play in the development of care packages to address neonatal mortality in the SDG era.

We computed summary baseline rates of immediate breastfeeding and facility births as well as percentage point changes due to the interventions. However, some authors did not include such information in their papers. While some reported baseline data, others only reported comparisons at endline. As a result the difference-in-difference between intervention and comparison groups could not be calculated. Thus improvements in newborn care practices are not adjusted for differences in baseline values between intervention and comparison groups for some studies. Moreover, our analysis was constrained by the lack of reporting on health system factors such as availability of health facilities or health providers. This reminds us of the value of a careful description of the context in which interventions are implemented to enable an understanding of the transferability of results.

In conclusion, the findings suggest that beyond a certain mortality threshold, community approaches alone might not lead to marked improvements in survival. This finding supports the recent trend in the SDG era towards increasing investment in the quality of facility care.
